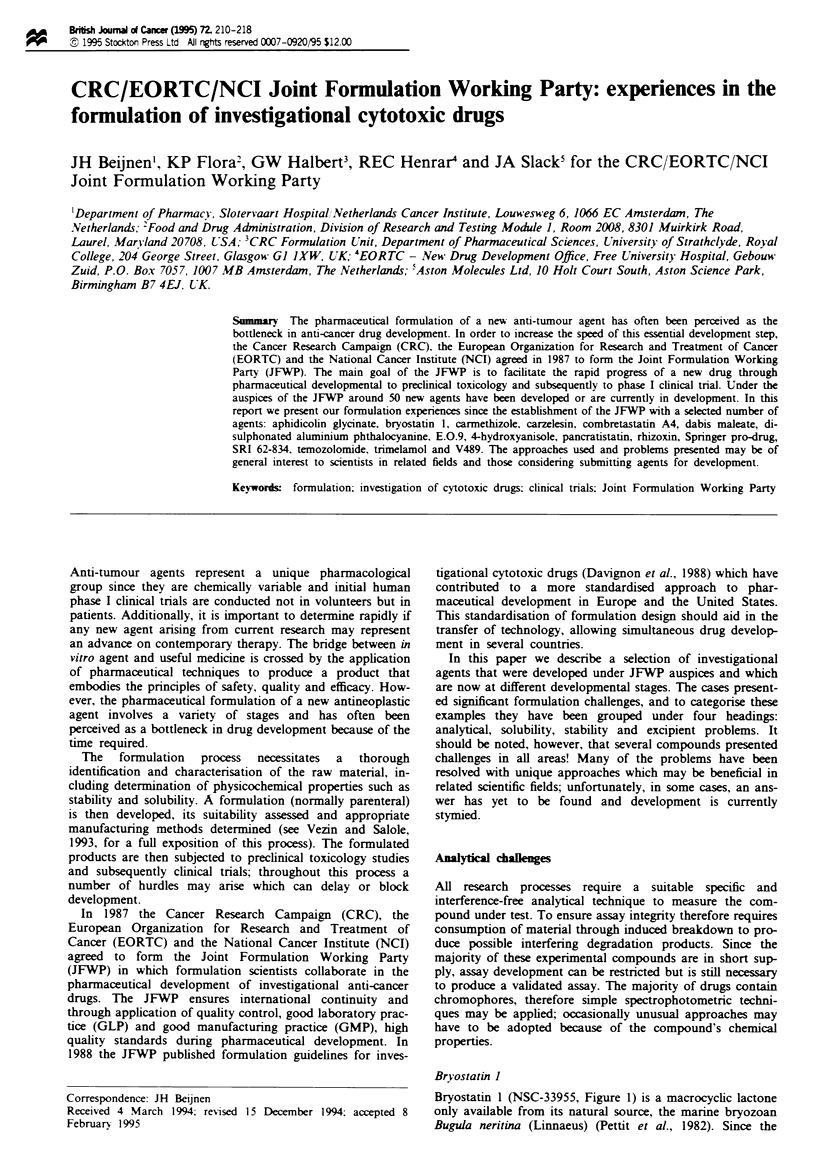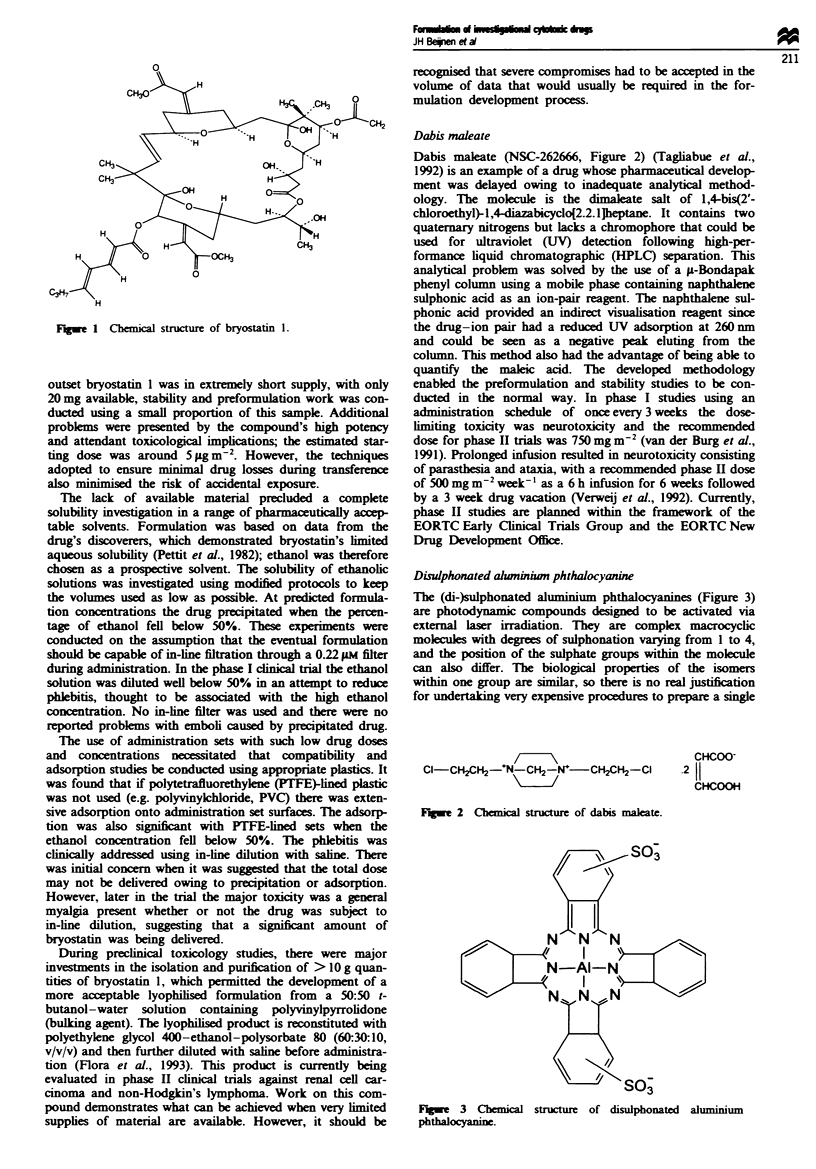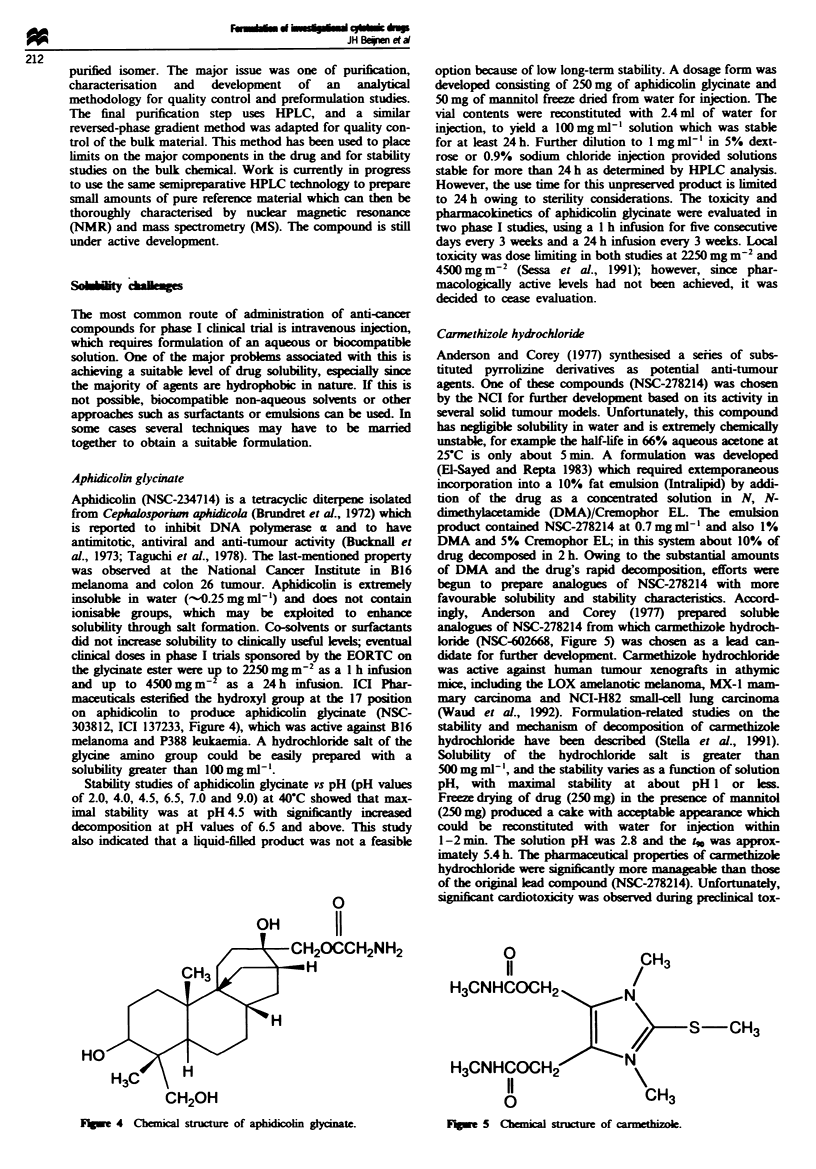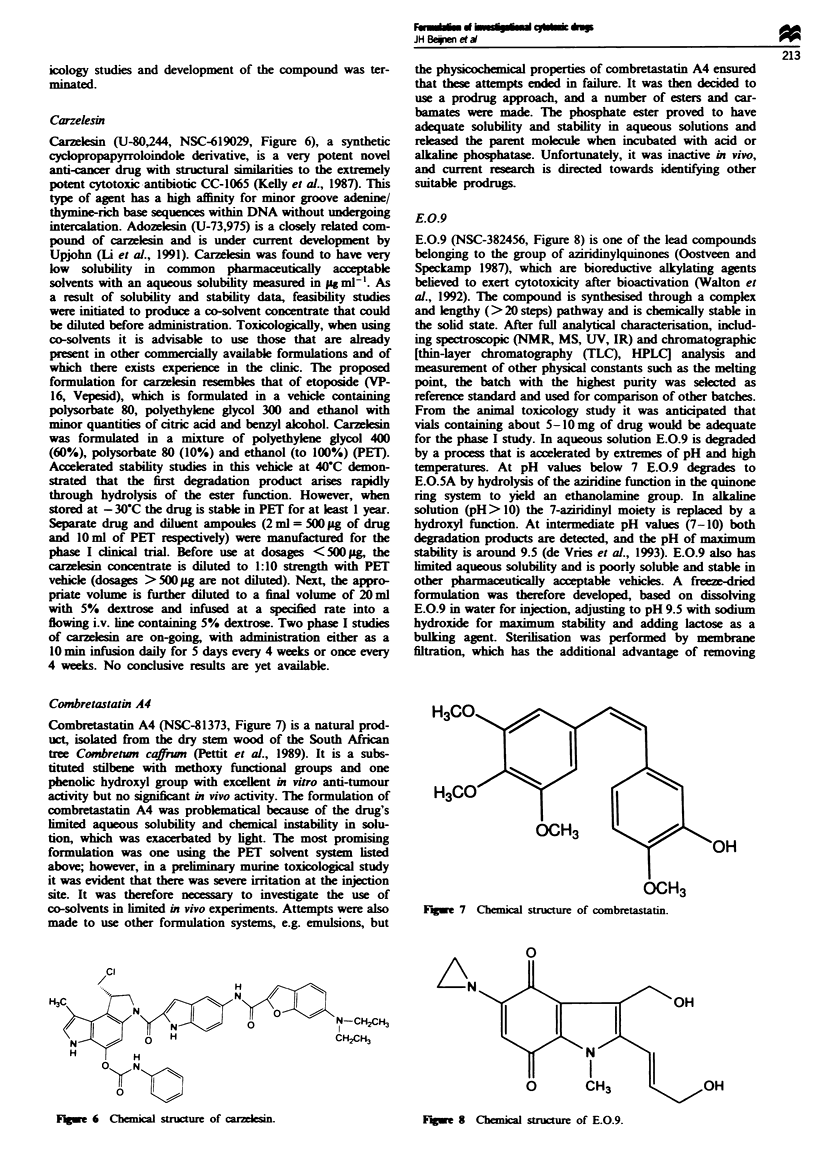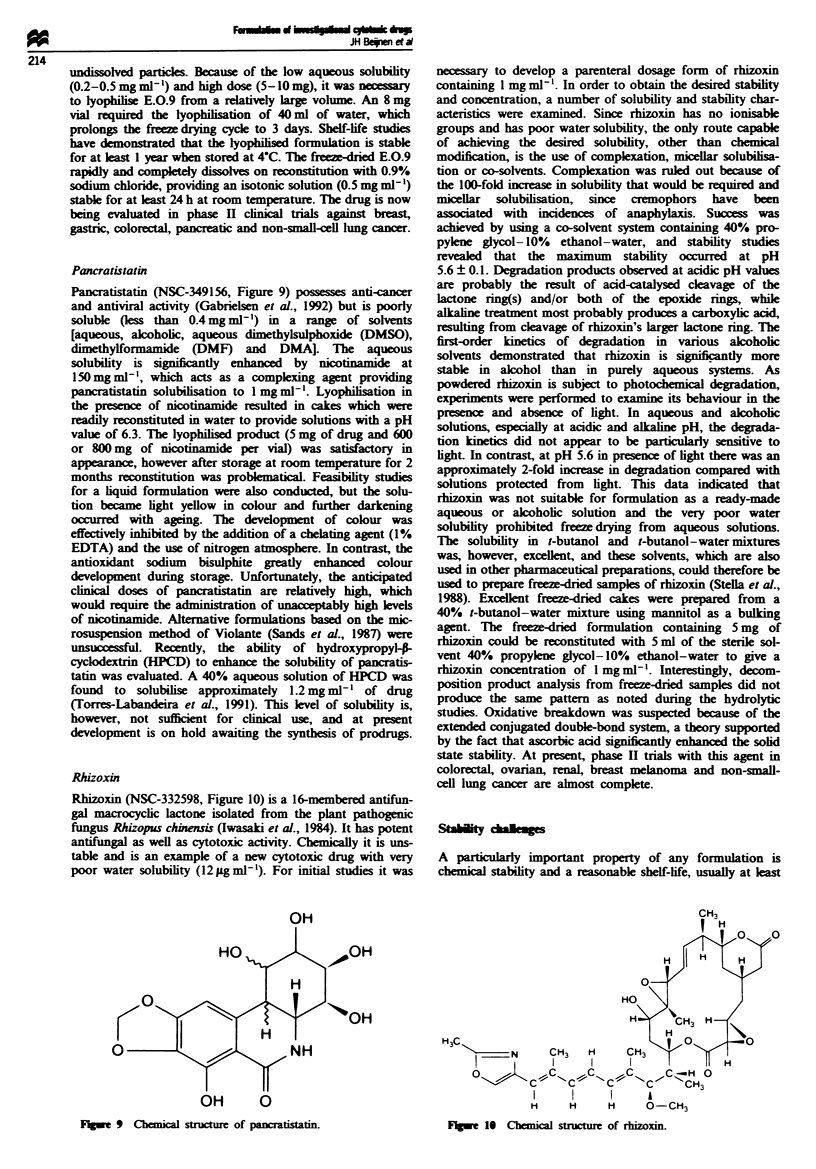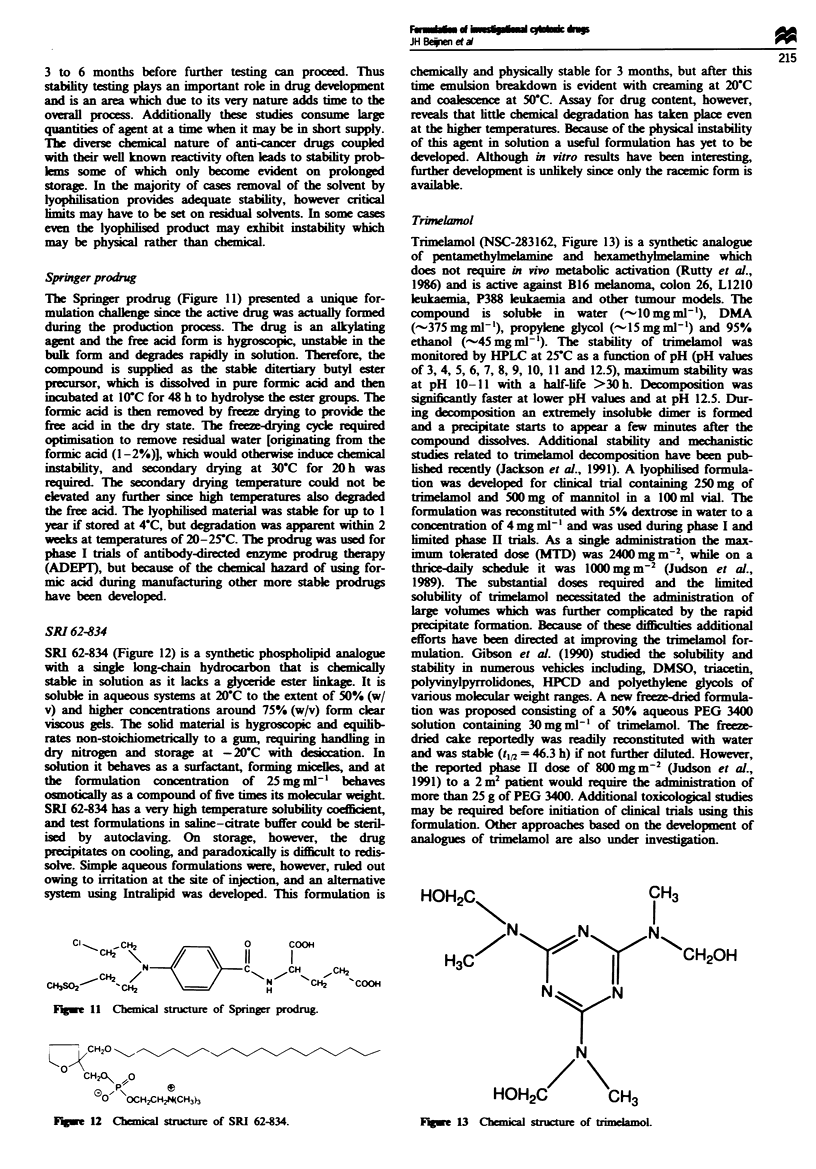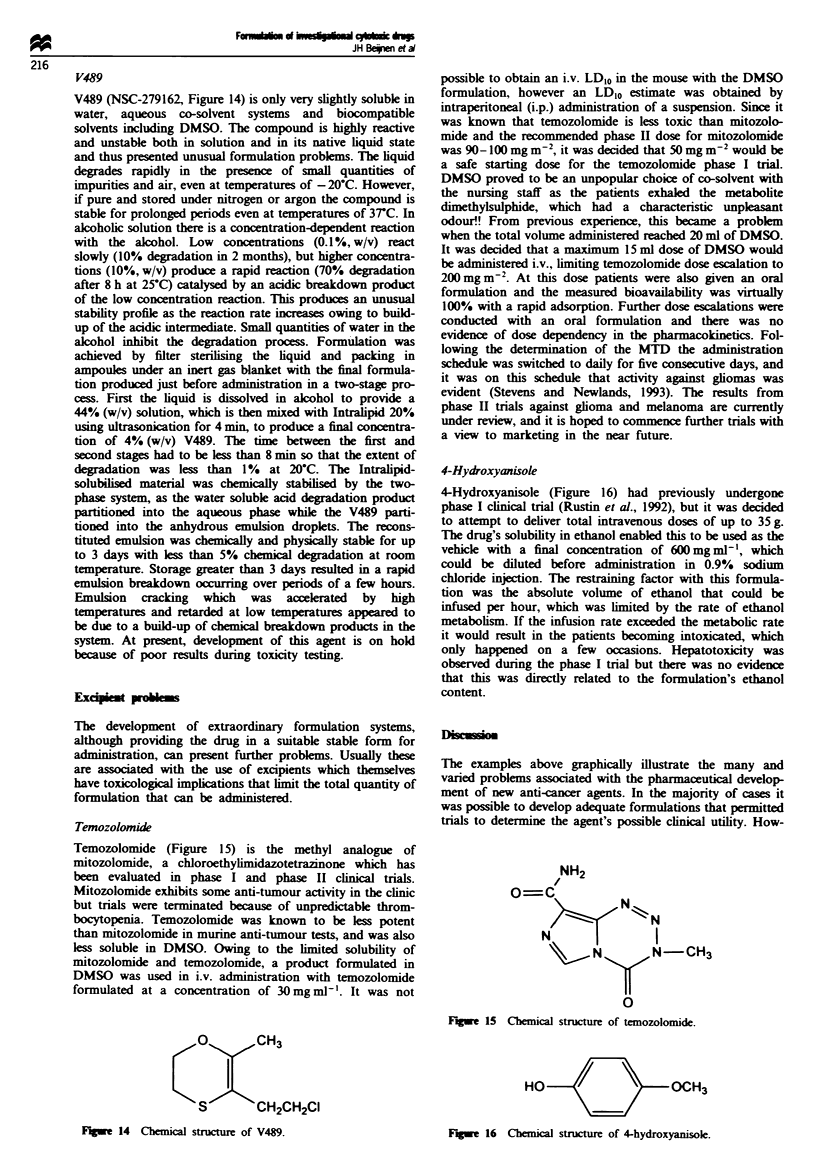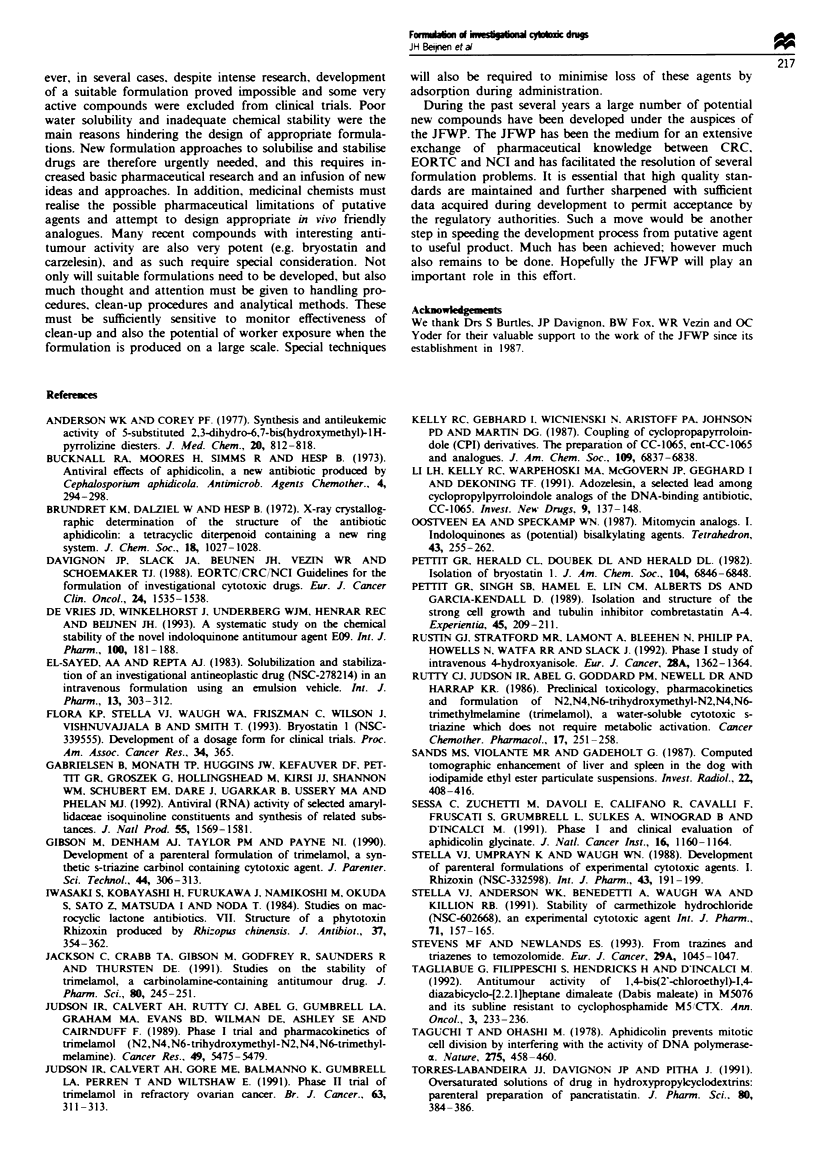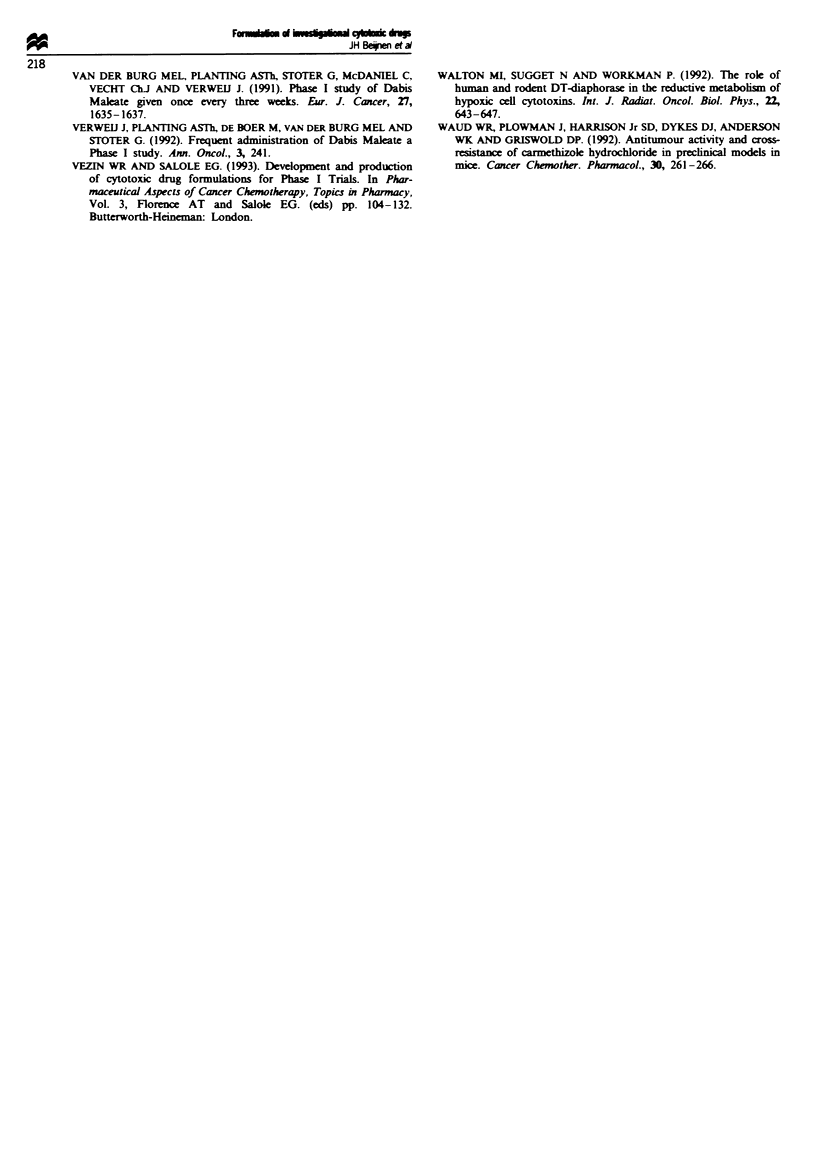# CRC/EORTC/NCI Joint Formulation Working Party: experiences in the formulation of investigational cytotoxic drugs.

**DOI:** 10.1038/bjc.1995.305

**Published:** 1995-07

**Authors:** J. H. Beijnen, K. P. Flora, G. W. Halbert, R. E. Henrar, J. A. Slack

**Affiliations:** Department of Pharmacy, Slotervaart Hospital/Netherlands Cancer Institute, Amsterdam.

## Abstract

The pharmaceutical formulation of a new anti-tumour agent has often been perceived as the bottleneck in anti-cancer drug development. In order to increase the speed of this essential development step, the Cancer Research Campaign (CRC), the European Organization for Research and Treatment of Cancer (EORTC) and the National Cancer Institute (NCI) agreed in 1987 to form the Joint Formulation Working Party (JFWP). The main goal of the JFWP is to facilitate the rapid progress of a new drug through pharmaceutical developmental to preclinical toxicology and subsequently to phase I clinical trial. Under the auspices of the JFWP around 50 new agents have been developed or are currently in development. In this report we present our formulation experiences since the establishment of the JFWP with a selected number of agents: aphidicolin glycinate, bryostatin 1, carmethizole, carzelesin, combretastatin A4, dabis maleate, disulphonated aluminium phthalocyanine, E.O.9, 4-hydroxyanisole, pancratistatin, rhizoxin, Springer pro-drug, SRI 62-834, temozolomide, trimelamol and V489. The approaches used and problems presented may be of general interest to scientists in related fields and those considering submitting agents for development.


British Journal of Cancer (1995) 72, 210-218

© 1995 Stockton Press Ltd All rights reserved 0007-0920/95 $12.00

### CRC/EORTC/NCI Joint Formulation Working Party: experiences in the formulation of investigational cytotoxic drugs

JH Beijnen[1], KP Flora[2], GW Halbert[3], REC Henrar[4] and JA Slack[5] for the CRC/EORTC/NCI Joint Formulation Working Party

[1]*Department of Pharmacy, Slotervaart Hospital/Netherlands Cancer Institute, Louwesweg 6, 1066 EC Amsterdam, The Netherlands;* [2]*Food and Drug Administration, Division of Research and Testing Module 1, Room 2008, 8301 Muirkirk Road, Laurel, Maryland 20708, USA;* [3]*CRC Formulation Unit, Department of Pharmaceutical Sciences, University of Strathclyde, Royal College, 204 George Street, Glasgow G1 1XW, UK;* [4]*EORTC – New Drug Development Office, Free University Hospital, Gebouw Zuid, P.O. Box 7057, 1007 MB Amsterdam, The Netherlands;* [5]*Aston Molecules Ltd, 10 Holt Court South, Aston Science Park, Birmingham B7 4EJ, UK.*

**Summary** The pharmaceutical formulation of a new anti-tumour agent has often been perceived as the bottleneck in anti-cancer drug development. In order to increase the speed of this essential development step, the Cancer Research Campaign (CRC), the European Organization for Research and Treatment of Cancer (EORTC) and the National Cancer Institute (NCI) agreed in 1987 to form the Joint Formulation Working Party (JFWP). The main goal of the JFWP is to facilitate the rapid progress of a new drug through pharmaceutical developmental to preclinical toxicology and subsequently to phase I clinical trial. Under the auspices of the JFWP around 50 new agents have been developed or are currently in development. In this report we present our formulation experiences since the establishment of the JFWP with a selected number of agents: aphidicolin glycinate, bryostatin 1, carmethizole, carzelesin, combretastatin A4, dabis maleate, disulphonated aluminium phthalocyanine, E.O.9, 4-hydroxyanisole, pancratistatin, rhizoxin, Springer pro-drug, SRI 62-834, temozolomide, trimelamol and V489. The approaches used and problems presented may be of general interest to scientists in related fields and those considering submitting agents for development.

**Keywords:** formulation; investigation of cytotoxic drugs; clinical trials; Joint Formulation Working Party

Anti-tumour agents represent a unique pharmacological group since they are chemically variable and initial human phase I clinical trials are conducted not in volunteers but in patients. Additionally, it is important to determine rapidly if any new agent arising from current research may represent an advance on contemporary therapy. The bridge between *in vitro* agent and useful medicine is crossed by the application of pharmaceutical techniques to produce a product that embodies the principles of safety, quality and efficacy. However, the pharmaceutical formulation of a new antineoplastic agent involves a variety of stages and has often been perceived as a bottleneck in drug development because of the time required.

The formulation process necessitates a thorough identification and characterisation of the raw material, including determination of physicochemical properties such as stability and solubility. A formulation (normally parenteral) is then developed, its suitability assessed and appropriate manufacturing methods determined (see Vezin and Salole, 1993, for a full exposition of this process). The formulated products are then subjected to preclinical toxicology studies and subsequently clinical trials; throughout this process a number of hurdles may arise which can delay or block development.

In 1987 the Cancer Research Campaign (CRC), the European Organization for Research and Treatment of Cancer (EORTC) and the National Cancer Institute (NCI) agreed to form the Joint Formulation Working Party (JFWP) in which formulation scientists collaborate in the pharmaceutical development of investigational anti-cancer drugs. The JFWP ensures international continuity and through application of quality control, good laboratory practice (GLP) and good manufacturing practice (GMP), high quality standards during pharmaceutical development. In 1988 the JFWP published formulation guidelines for investigational cytotoxic drugs (Davignon *et al.*, 1988) which have contributed to a more standardised approach to pharmaceutical development in Europe and the United States. This standardisation of formulation design should aid in the transfer of technology, allowing simultaneous drug development in several countries.

In this paper we describe a selection of investigational agents that were developed under JFWP auspices and which are now at different developmental stages. The cases presented significant formulation challenges, and to categorise these examples they have been grouped under four headings: analytical, solubility, stability and excipient problems. It should be noted, however, that several compounds presented challenges in all areas! Many of the problems have been resolved with unique approaches which may be beneficial in related scientific fields; unfortunately, in some cases, an answer has yet to be found and development is currently stymied.

### Analytical challenges

All research processes require a suitable specific and interference-free analytical technique to measure the compound under test. To ensure assay integrity therefore requires consumption of material through induced breakdown to produce possible interfering degradation products. Since the majority of these experimental compounds are in short supply, assay development can be restricted but is still necessary to produce a validated assay. The majority of drugs contain chromophores, therefore simple spectrophotometric techniques may be applied; occasionally unusual approaches may have to be adopted because of the compound's chemical properties.

### *Bryostatin 1*

Bryostatin 1 (NSC-33955, Figure 1) is a macrocyclic lactone only available from its natural source, the marine bryozoan *Bugula neritina* (Linnaeus) (Pettit *et al.*, 1982). Since the

Correspondence: JH Beijnen
Received 4 March 1994; revised 15 December 1994; accepted 8 February 1995

**Figure 1** Chemical structure of bryostatin 1.

outset bryostatin 1 was in extremely short supply, with only 20 mg available, stability and preformulation work was conducted using a small proportion of this sample. Additional problems were presented by the compound's high potency and attendant toxicological implications; the estimated starting dose was around 5 $\mu g\ m^{-2}$. However, the techniques adopted to ensure minimal drug losses during transference also minimised the risk of accidental exposure.

The lack of available material precluded a complete solubility investigation in a range of pharmaceutically acceptable solvents. Formulation was based on data from the drug's discoverers, which demonstrated bryostatin's limited aqueous solubility (Pettit *et al.*, 1982); ethanol was therefore chosen as a prospective solvent. The solubility of ethanolic solutions was investigated using modified protocols to keep the volumes used as low as possible. At predicted formulation concentrations the drug precipitated when the percentage of ethanol fell below 50%. These experiments were conducted on the assumption that the eventual formulation should be capable of in-line filtration through a 0.22 $\mu$M filter during administration. In the phase I clinical trial the ethanol solution was diluted well below 50% in an attempt to reduce phlebitis, thought to be associated with the high ethanol concentration. No in-line filter was used and there were no reported problems with emboli caused by precipitated drug.

The use of administration sets with such low drug doses and concentrations necessitated that compatibility and adsorption studies be conducted using appropriate plastics. It was found that if polytetrafluorethylene (PTFE)-lined plastic was not used (e.g. polyvinylchloride, PVC) there was extensive adsorption onto administration set surfaces. The adsorption was also significant with PTFE-lined sets when the ethanol concentration fell below 50%. The phlebitis was clinically addressed using in-line dilution with saline. There was initial concern when it was suggested that the total dose may not be delivered owing to precipitation or adsorption. However, later in the trial the major toxicity was a general myalgia present whether or not the drug was subject to in-line dilution, suggesting that a significant amount of bryostatin was being delivered.

During preclinical toxicology studies, there were major investments in the isolation and purification of > 10 g quantities of bryostatin 1, which permitted the development of a more acceptable lyophilised formulation from a 50:50 *t*-butanol–water solution containing polyvinylpyrrolidone (bulking agent). The lyophilised product is reconstituted with polyethylene glycol 400–ethanol–polysorbate 80 (60:30:10, v/v/v) and then further diluted with saline before administration (Flora *et al.*, 1993). This product is currently being evaluated in phase II clinical trials against renal cell carcinoma and non-Hodgkin's lymphoma. Work on this compound demonstrates what can be achieved when very limited supplies of material are available. However, it should be recognised that severe compromises had to be accepted in the volume of data that would usually be required in the formulation development process.

### *Dabis maleate*

Dabis maleate (NSC-262666, Figure 2) (Tagliabue *et al.*, 1992) is an example of a drug whose pharmaceutical development was delayed owing to inadequate analytical methodology. The molecule is the dimaleate salt of 1,4-bis(2'-chloroethyl)-1,4-diazabicyclo[2.2.1]heptane. It contains two quaternary nitrogens but lacks a chromophore that could be used for ultraviolet (UV) detection following high-performance liquid chromatographic (HPLC) separation. This analytical problem was solved by the use of a $\mu$-Bondapak phenyl column using a mobile phase containing naphthalene sulphonic acid as an ion-pair reagent. The naphthalene sulphonic acid provided an indirect visualisation reagent since the drug–ion pair had a reduced UV adsorption at 260 nm and could be seen as a negative peak eluting from the column. This method also had the advantage of being able to quantify the maleic acid. The developed methodology enabled the preformulation and stability studies to be conducted in the normal way. In phase I studies using an administration schedule of once every 3 weeks the dose-limiting toxicity was neurotoxicity and the recommended dose for phase II trials was 750 mg $m^{-2}$ (van der Burg *et al.*, 1991). Prolonged infusion resulted in neurotoxicity consisting of parasthesia and ataxia, with a recommended phase II dose of 500 mg $m^{-2}$ $week^{-1}$ as a 6 h infusion for 6 weeks followed by a 3 week drug vacation (Verweij *et al.*, 1992). Currently, phase II studies are planned within the framework of the EORTC Early Clinical Trials Group and the EORTC New Drug Development Office.

### *Disulphonated aluminium phthalocyanine*

The (di-)sulphonated aluminium phthalocyanines (Figure 3) are photodynamic compounds designed to be activated via external laser irradiation. They are complex macrocyclic molecules with degrees of sulphonation varying from 1 to 4, and the position of the sulphate groups within the molecule can also differ. The biological properties of the isomers within one group are similar, so there is no real justification for undertaking very expensive procedures to prepare a single

**Figure 2** Chemical structure of dabis maleate.

**Figure 3** Chemical structure of disulphonated aluminium phthalocyanine.

purified isomer. The major issue was one of purification, characterisation and development of an analytical methodology for quality control and preformulation studies. The final purification step uses HPLC, and a similar reversed-phase gradient method was adapted for quality control of the bulk material. This method has been used to place limits on the major components in the drug and for stability studies on the bulk chemical. Work is currently in progress to use the same semipreparative HPLC technology to prepare small amounts of pure reference material which can then be thoroughly characterised by nuclear magnetic resonance (NMR) and mass spectrometry (MS). The compound is still under active development.

### Solubility challenges

The most common route of administration of anti-cancer compounds for phase I clinical trial is intravenous injection, which requires formulation of an aqueous or biocompatible solution. One of the major problems associated with this is achieving a suitable level of drug solubility, especially since the majority of agents are hydrophobic in nature. If this is not possible, biocompatible non-aqueous solvents or other approaches such as surfactants or emulsions can be used. In some cases several techniques may have to be married together to obtain a suitable formulation.

### *Aphidicolin glycinate*

Aphidicolin (NSC-234714) is a tetracyclic diterpene isolated from *Cephalosporium aphidicola* (Brundret *et al*., 1972) which is reported to inhibit DNA polymerase α and to have antimitotic, antiviral and anti-tumour activity (Bucknall *et al*., 1973; Taguchi *et al*., 1978). The last-mentioned property was observed at the National Cancer Institute in B16 melanoma and colon 26 tumour. Aphidicolin is extremely insoluble in water (~0.25 mg ml$^{-1}$) and does not contain ionisable groups, which may be exploited to enhance solubility through salt formation. Co-solvents or surfactants did not increase solubility to clinically useful levels; eventual clinical doses in phase I trials sponsored by the EORTC on the glycinate ester were up to 2250 mg m$^{-2}$ as a 1 h infusion and up to 4500 mg m$^{-2}$ as a 24 h infusion. ICI Pharmaceuticals esterified the hydroxyl group at the 17 position on aphidicolin to produce aphidicolin glycinate (NSC-303812, ICI 137233, Figure 4), which was active against B16 melanoma and P388 leukaemia. A hydrochloride salt of the glycine amino group could be easily prepared with a solubility greater than 100 mg ml$^{-1}$.

Stability studies of aphidicolin glycinate *vs* pH (pH values of 2.0, 4.0, 4.5, 6.5, 7.0 and 9.0) at 40°C showed that maximal stability was at pH 4.5 with significantly increased decomposition at pH values of 6.5 and above. This study also indicated that a liquid-filled product was not a feasible option because of low long-term stability. A dosage form was developed consisting of 250 mg of aphidicolin glycinate and 50 mg of mannitol freeze dried from water for injection. The vial contents were reconstituted with 2.4 ml of water for injection, to yield a 100 mg ml$^{-1}$ solution which was stable for at least 24 h. Further dilution to 1 mg ml$^{-1}$ in 5% dextrose or 0.9% sodium chloride injection provided solutions stable for more than 24 h as determined by HPLC analysis. However, the use time for this unpreserved product is limited to 24 h owing to sterility considerations. The toxicity and pharmacokinetics of aphidicolin glycinate were evaluated in two phase I studies, using a 1 h infusion for five consecutive days every 3 weeks and a 24 h infusion every 3 weeks. Local toxicity was dose limiting in both studies at 2250 mg m$^{-2}$ and 4500 mg m$^{-2}$ (Sessa *et al*., 1991); however, since pharmacologically active levels had not been achieved, it was decided to cease evaluation.

### *Carmethizole hydrochloride*

Anderson and Corey (1977) synthesised a series of substituted pyrrolizine derivatives as potential anti-tumour agents. One of these compounds (NSC-278214) was chosen by the NCI for further development based on its activity in several solid tumour models. Unfortunately, this compound has negligible solubility in water and is extremely chemically unstable, for example the half-life in 66% aqueous acetone at 25°C is only about 5 min. A formulation was developed (El-Sayed and Repta 1983) which required extemporaneous incorporation into a 10% fat emulsion (Intralipid) by addition of the drug as a concentrated solution in *N*, *N*-dimethylacetamide (DMA)/Cremophor EL. The emulsion product contained NSC-278214 at 0.7 mg ml$^{-1}$ and also 1% DMA and 5% Cremophor EL; in this system about 10% of drug decomposed in 2 h. Owing to the substantial amounts of DMA and the drug's rapid decomposition, efforts were begun to prepare analogues of NSC-278214 with more favourable solubility and stability characteristics. Accordingly, Anderson and Corey (1977) prepared soluble analogues of NSC-278214 from which carmethizole hydrochloride (NSC-602668, Figure 5) was chosen as a lead candidate for further development. Carmethizole hydrochloride was active against human tumour xenografts in athymic mice, including the LOX amelanotic melanoma, MX-1 mammary carcinoma and NCI-H82 small-cell lung carcinoma (Waud *et al*., 1992). Formulation-related studies on the stability and mechanism of decomposition of carmethizole hydrochloride have been described (Stella *et al*., 1991). Solubility of the hydrochloride salt is greater than 500 mg ml$^{-1}$, and the stability varies as a function of solution pH, with maximal stability at about pH 1 or less. Freeze drying of drug (250 mg) in the presence of mannitol (250 mg) produced a cake with acceptable appearance which could be reconstituted with water for injection within 1–2 min. The solution pH was 2.8 and the $t_{90}$ was approximately 5.4 h. The pharmaceutical properties of carmethizole hydrochloride were significantly more manageable than those of the original lead compound (NSC-278214). Unfortunately, significant cardiotoxicity was observed during preclinical tox-

**Figure 4** Chemical structure of aphidicolin glycinate.

**Figure 5** Chemical structure of carmethizole.

icology studies and development of the compound was terminated.

### *Carzelesin*

Carzelesin (U-80,244, NSC-619029, Figure 6), a synthetic cyclopropapyrroloindole derivative, is a very potent novel anti-cancer drug with structural similarities to the extremely potent cytotoxic antibiotic CC-1065 (Kelly *et al.*, 1987). This type of agent has a high affinity for minor groove adenine/thymine-rich base sequences within DNA without undergoing intercalation. Adozelesin (U-73,975) is a closely related compound of carzelesin and is under current development by Upjohn (Li *et al.*, 1991). Carzelesin was found to have very low solubility in common pharmaceutically acceptable solvents with an aqueous solubility measured in $\mu g\ ml^{-1}$. As a result of solubility and stability data, feasibility studies were initiated to produce a co-solvent concentrate that could be diluted before administration. Toxicologically, when using co-solvents it is advisable to use those that are already present in other commercially available formulations and of which there exists experience in the clinic. The proposed formulation for carzelesin resembles that of etoposide (VP-16, Vepesid), which is formulated in a vehicle containing polysorbate 80, polyethylene glycol 300 and ethanol with minor quantities of citric acid and benzyl alcohol. Carzelesin was formulated in a mixture of polyethylene glycol 400 (60%), polysorbate 80 (10%) and ethanol (to 100%) (PET). Accelerated stability studies in this vehicle at 40°C demonstrated that the first degradation product arises rapidly through hydrolysis of the ester function. However, when stored at −30°C the drug is stable in PET for at least 1 year. Separate drug and diluent ampoules (2 ml = 500 μg of drug and 10 ml of PET respectively) were manufactured for the phase I clinical trial. Before use at dosages < 500 μg, the carzelesin concentrate is diluted to 1:10 strength with PET vehicle (dosages > 500 μg are not diluted). Next, the appropriate volume is further diluted to a final volume of 20 ml with 5% dextrose and infused at a specified rate into a flowing i.v. line containing 5% dextrose. Two phase I studies of carzelesin are on-going, with administration either as a 10 min infusion daily for 5 days every 4 weeks or once every 4 weeks. No conclusive results are yet available.

### *Combretastatin A4*

Combretastatin A4 (NSC-81373, Figure 7) is a natural product, isolated from the dry stem wood of the South African tree *Combretum caffrum* (Pettit *et al.*, 1989). It is a substituted stilbene with methoxy functional groups and one phenolic hydroxyl group with excellent *in vitro* anti-tumour activity but no significant *in vivo* activity. The formulation of combretastatin A4 was problematical because of the drug's limited aqueous solubility and chemical instability in solution, which was exacerbated by light. The most promising formulation was one using the PET solvent system listed above; however, in a preliminary murine toxicological study it was evident that there was severe irritation at the injection site. It was therefore necessary to investigate the use of co-solvents in limited *in vivo* experiments. Attempts were also made to use other formulation systems, e.g. emulsions, but the physicochemical properties of combretastatin A4 ensured that these attempts ended in failure. It was then decided to use a prodrug approach, and a number of esters and carbamates were made. The phosphate ester proved to have adequate solubility and stability in aqueous solutions and released the parent molecule when incubated with acid or alkaline phosphatase. Unfortunately, it was inactive *in vivo*, and current research is directed towards identifying other suitable prodrugs.

### *E.O.9*

E.O.9 (NSC-382456, Figure 8) is one of the lead compounds belonging to the group of aziridinylquinones (Oostveen and Speckamp 1987), which are bioreductive alkylating agents believed to exert cytotoxicity after bioactivation (Walton *et al.*, 1992). The compound is synthesised through a complex and lengthy (> 20 steps) pathway and is chemically stable in the solid state. After full analytical characterisation, including spectroscopic (NMR, MS, UV, IR) and chromatographic [thin-layer chromatography (TLC), HPLC] analysis and measurement of other physical constants such as the melting point, the batch with the highest purity was selected as reference standard and used for comparison of other batches. From the animal toxicology study it was anticipated that vials containing about 5–10 mg of drug would be adequate for the phase I study. In aqueous solution E.O.9 is degraded by a process that is accelerated by extremes of pH and high temperatures. At pH values below 7 E.O.9 degrades to E.O.5A by hydrolysis of the aziridine function in the quinone ring system to yield an ethanolamine group. In alkaline solution (pH > 10) the 7-aziridinyl moiety is replaced by a hydroxyl function. At intermediate pH values (7–10) both degradation products are detected, and the pH of maximum stability is around 9.5 (de Vries *et al.*, 1993). E.O.9 also has limited aqueous solubility and is poorly soluble and stable in other pharmaceutically acceptable vehicles. A freeze-dried formulation was therefore developed, based on dissolving E.O.9 in water for injection, adjusting to pH 9.5 with sodium hydroxide for maximum stability and adding lactose as a bulking agent. Sterilisation was performed by membrane filtration, which has the additional advantage of removing

**Figure 6** Chemical structure of carzelesin.

**Figure 7** Chemical structure of combretastatin.

**Figure 8** Chemical structure of E.O.9.

undissolved particles. Because of the low aqueous solubility (0.2–0.5 mg $ml^{-1}$) and high dose (5–10 mg), it was necessary to lyophilise E.O.9 from a relatively large volume. An 8 mg vial required the lyophilisation of 40 ml of water, which prolongs the freeze drying cycle to 3 days. Shelf-life studies have demonstrated that the lyophilised formulation is stable for at least 1 year when stored at 4°C. The freeze-dried E.O.9 rapidly and completely dissolves on reconstitution with 0.9% sodium chloride, providing an isotonic solution (0.5 mg $ml^{-1}$) stable for at least 24 h at room temperature. The drug is now being evaluated in phase II clinical trials against breast, gastric, colorectal, pancreatic and non-small-cell lung cancer.

### *Pancratistatin*

Pancratistatin (NSC-349156, Figure 9) possesses anti-cancer and antiviral activity (Gabrielsen *et al.*, 1992) but is poorly soluble (less than 0.4 mg $ml^{-1}$) in a range of solvents [aqueous, alcoholic, aqueous dimethylsulphoxide (DMSO), dimethylformamide (DMF) and DMA]. The aqueous solubility is significantly enhanced by nicotinamide at 150 mg $ml^{-1}$, which acts as a complexing agent providing pancratistatin solubilisation to 1 mg $ml^{-1}$. Lyophilisation in the presence of nicotinamide resulted in cakes which were readily reconstituted in water to provide solutions with a pH value of 6.3. The lyophilised product (5 mg of drug and 600 or 800 mg of nicotinamide per vial) was satisfactory in appearance, however after storage at room temperature for 2 months reconstitution was problematical. Feasibility studies for a liquid formulation were also conducted, but the solution became light yellow in colour and further darkening occurred with ageing. The development of colour was effectively inhibited by the addition of a chelating agent (1% EDTA) and the use of nitrogen atmosphere. In contrast, the antioxidant sodium bisulphite greatly enhanced colour development during storage. Unfortunately, the anticipated clinical doses of pancratistatin are relatively high, which would require the administration of unacceptably high levels of nicotinamide. Alternative formulations based on the microsuspension method of Violante (Sands *et al.*, 1987) were unsuccessful. Recently, the ability of hydroxypropyl-β-cyclodextrin (HPCD) to enhance the solubility of pancratistatin was evaluated. A 40% aqueous solution of HPCD was found to solubilise approximately 1.2 mg $ml^{-1}$ of drug (Torres-Labandeira *et al.*, 1991). This level of solubility is, however, not sufficient for clinical use, and at present development is on hold awaiting the synthesis of prodrugs.

### *Rhizoxin*

Rhizoxin (NSC-332598, Figure 10) is a 16-membered antifungal macrocyclic lactone isolated from the plant pathogenic fungus *Rhizopus chinensis* (Iwasaki *et al.*, 1984). It has potent antifungal as well as cytotoxic activity. Chemically it is unstable and is an example of a new cytotoxic drug with very poor water solubility (12 μg $ml^{-1}$). For initial studies it was necessary to develop a parenteral dosage form of rhizoxin containing 1 mg $ml^{-1}$. In order to obtain the desired stability and concentration, a number of solubility and stability characteristics were examined. Since rhizoxin has no ionisable groups and has poor water solubility, the only route capable of achieving the desired solubility, other than chemical modification, is the use of complexation, micellar solubilisation or co-solvents. Complexation was ruled out because of the 100-fold increase in solubility that would be required and micellar solubilisation, since cremophors have been associated with incidences of anaphylaxis. Success was achieved by using a co-solvent system containing 40% propylene glycol–10% ethanol–water, and stability studies revealed that the maximum stability occurred at pH 5.6 ± 0.1. Degradation products observed at acidic pH values are probably the result of acid-catalysed cleavage of the lactone ring(s) and/or both of the epoxide rings, while alkaline treatment most probably produces a carboxylic acid, resulting from cleavage of rhizoxin's larger lactone ring. The first-order kinetics of degradation in various alcoholic solvents demonstrated that rhizoxin is significantly more stable in alcohol than in purely aqueous systems. As powdered rhizoxin is subject to photochemical degradation, experiments were performed to examine its behaviour in the presence and absence of light. In aqueous and alcoholic solutions, especially at acidic and alkaline pH, the degradation kinetics did not appear to be particularly sensitive to light. In contrast, at pH 5.6 in presence of light there was an approximately 2-fold increase in degradation compared with solutions protected from light. This data indicated that rhizoxin was not suitable for formulation as a ready-made aqueous or alcoholic solution and the very poor water solubility prohibited freeze drying from aqueous solutions. The solubility in *t*-butanol and *t*-butanol–water mixtures was, however, excellent, and these solvents, which are also used in other pharmaceutical preparations, could therefore be used to prepare freeze-dried samples of rhizoxin (Stella *et al.*, 1988). Excellent freeze-dried cakes were prepared from a 40% *t*-butanol–water mixture using mannitol as a bulking agent. The freeze-dried formulation containing 5 mg of rhizoxin could be reconstituted with 5 ml of the sterile solvent 40% propylene glycol–10% ethanol–water to give a rhizoxin concentration of 1 mg $ml^{-1}$. Interestingly, decomposition product analysis from freeze-dried samples did not produce the same pattern as noted during the hydrolytic studies. Oxidative breakdown was suspected because of the extended conjugated double-bond system, a theory supported by the fact that ascorbic acid significantly enhanced the solid state stability. At present, phase II trials with this agent in colorectal, ovarian, renal, breast melanoma and non-small-cell lung cancer are almost complete.

### Stability challenges

A particularly important property of any formulation is chemical stability and a reasonable shelf-life, usually at least

**Figure 9** Chemical structure of pancratistatin.

**Figure 10** Chemical structure of rhizoxin.

3 to 6 months before further testing can proceed. Thus stability testing plays an important role in drug development and is an area which due to its very nature adds time to the overall process. Additionally these studies consume large quantities of agent at a time when it may be in short supply. The diverse chemical nature of anti-cancer drugs coupled with their well known reactivity often leads to stability problems some of which only become evident on prolonged storage. In the majority of cases removal of the solvent by lyophilisation provides adequate stability, however critical limits may have to be set on residual solvents. In some cases even the lyophilised product may exhibit instability which may be physical rather than chemical.

### *Springer prodrug*

The Springer prodrug (Figure 11) presented a unique formulation challenge since the active drug was actually formed during the production process. The drug is an alkylating agent and the free acid form is hygroscopic, unstable in the bulk form and degrades rapidly in solution. Therefore, the compound is supplied as the stable ditertiary butyl ester precursor, which is dissolved in pure formic acid and then incubated at 10°C for 48 h to hydrolyse the ester groups. The formic acid is then removed by freeze drying to provide the free acid in the dry state. The freeze-drying cycle required optimisation to remove residual water [originating from the formic acid (1–2%)], which would otherwise induce chemical instability, and secondary drying at 30°C for 20 h was required. The secondary drying temperature could not be elevated any further since high temperatures also degraded the free acid. The lyophilised material was stable for up to 1 year if stored at 4°C, but degradation was apparent within 2 weeks at temperatures of 20–25°C. The prodrug was used for phase I trials of antibody-directed enzyme prodrug therapy (ADEPT), but because of the chemical hazard of using formic acid during manufacturing other more stable prodrugs have been developed.

### *SRI 62-834*

SRI 62-834 (Figure 12) is a synthetic phospholipid analogue with a single long-chain hydrocarbon that is chemically stable in solution as it lacks a glyceride ester linkage. It is soluble in aqueous systems at 20°C to the extent of 50% (w/v) and higher concentrations around 75% (w/v) form clear viscous gels. The solid material is hygroscopic and equilibrates non-stoichiometrically to a gum, requiring handling in dry nitrogen and storage at −20°C with desiccation. In solution it behaves as a surfactant, forming micelles, and at the formulation concentration of 25 mg ml$^{-1}$ behaves osmotically as a compound of five times its molecular weight. SRI 62-834 has a very high temperature solubility coefficient, and test formulations in saline–citrate buffer could be sterilised by autoclaving. On storage, however, the drug precipitates on cooling, and paradoxically is difficult to redissolve. Simple aqueous formulations were, however, ruled out owing to irritation at the site of injection, and an alternative system using Intralipid was developed. This formulation is chemically and physically stable for 3 months, but after this time emulsion breakdown is evident with creaming at 20°C and coalescence at 50°C. Assay for drug content, however, reveals that little chemical degradation has taken place even at the higher temperatures. Because of the physical instability of this agent in solution a useful formulation has yet to be developed. Although *in vitro* results have been interesting, further development is unlikely since only the racemic form is available.

**Figure 11** Chemical structure of Springer prodrug.

**Figure 12** Chemical structure of SRI 62-834.

### *Trimelamol*

Trimelamol (NSC-283162, Figure 13) is a synthetic analogue of pentamethylmelamine and hexamethylmelamine which does not require *in vivo* metabolic activation (Rutty *et al.*, 1986) and is active against B16 melanoma, colon 26, L1210 leukaemia, P388 leukaemia and other tumour models. The compound is soluble in water (~10 mg ml$^{-1}$), DMA (~375 mg ml$^{-1}$), propylene glycol (~15 mg ml$^{-1}$) and 95% ethanol (~45 mg ml$^{-1}$). The stability of trimelamol was monitored by HPLC at 25°C as a function of pH (pH values of 3, 4, 5, 6, 7, 8, 9, 10, 11 and 12.5), maximum stability was at pH 10–11 with a half-life >30 h. Decomposition was significantly faster at lower pH values and at pH 12.5. During decomposition an extremely insoluble dimer is formed and a precipitate starts to appear a few minutes after the compound dissolves. Additional stability and mechanistic studies related to trimelamol decomposition have been published recently (Jackson *et al.*, 1991). A lyophilised formulation was developed for clinical trial containing 250 mg of trimelamol and 500 mg of mannitol in a 100 ml vial. The formulation was reconstituted with 5% dextrose in water to a concentration of 4 mg ml$^{-1}$ and was used during phase I and limited phase II trials. As a single administration the maximum tolerated dose (MTD) was 2400 mg m$^{-2}$, while on a thrice-daily schedule it was 1000 mg m$^{-2}$ (Judson *et al.*, 1989). The substantial doses required and the limited solubility of trimelamol necessitated the administration of large volumes which was further complicated by the rapid precipitate formation. Because of these difficulties additional efforts have been directed at improving the trimelamol formulation. Gibson *et al.* (1990) studied the solubility and stability in numerous vehicles including, DMSO, triacetin, polyvinylpyrrolidones, HPCD and polyethylene glycols of various molecular weight ranges. A new freeze-dried formulation was proposed consisting of a 50% aqueous PEG 3400 solution containing 30 mg ml$^{-1}$ of trimelamol. The freeze-dried cake reportedly was readily reconstituted with water and was stable ($t_{1/2}$ = 46.3 h) if not further diluted. However, the reported phase II dose of 800 mg m$^{-2}$ (Judson *et al.*, 1991) to a 2 m$^2$ patient would require the administration of more than 25 g of PEG 3400. Additional toxicological studies may be required before initiation of clinical trials using this formulation. Other approaches based on the development of analogues of trimelamol are also under investigation.

**Figure 13** Chemical structure of trimelamol.

*V489*

V489 (NSC-279162, Figure 14) is only very slightly soluble in water, aqueous co-solvent systems and biocompatible solvents including DMSO. The compound is highly reactive and unstable both in solution and in its native liquid state and thus presented unusual formulation problems. The liquid degrades rapidly in the presence of small quantities of impurities and air, even at temperatures of –20°C. However, if pure and stored under nitrogen or argon the compound is stable for prolonged periods even at temperatures of 37°C. In alcoholic solution there is a concentration-dependent reaction with the alcohol. Low concentrations (0.1%, w/v) react slowly (10% degradation in 2 months), but higher concentrations (10%, w/v) produce a rapid reaction (70% degradation after 8 h at 25°C) catalysed by an acidic breakdown product of the low concentration reaction. This produces an unusual stability profile as the reaction rate increases owing to build-up of the acidic intermediate. Small quantities of water in the alcohol inhibit the degradation process. Formulation was achieved by filter sterilising the liquid and packing in ampoules under an inert gas blanket with the final formulation produced just before administration in a two-stage process. First the liquid is dissolved in alcohol to provide a 44% (w/v) solution, which is then mixed with Intralipid 20% using ultrasonication for 4 min, to produce a final concentration of 4% (w/v) V489. The time between the first and second stages had to be less than 8 min so that the extent of degradation was less than 1% at 20°C. The Intralipid-solubilised material was chemically stabilised by the two-phase system, as the water soluble acid degradation product partitioned into the aqueous phase while the V489 partitioned into the anhydrous emulsion droplets. The reconstituted emulsion was chemically and physically stable for up to 3 days with less than 5% chemical degradation at room temperature. Storage greater than 3 days resulted in a rapid emulsion breakdown occurring over periods of a few hours. Emulsion cracking which was accelerated by high temperatures and retarded at low temperatures appeared to be due to a build-up of chemical breakdown products in the system. At present, development of this agent is on hold because of poor results during toxicity testing.

### Excipient problems

The development of extraordinary formulation systems, although providing the drug in a suitable stable form for administration, can present further problems. Usually these are associated with the use of excipients which themselves have toxicological implications that limit the total quantity of formulation that can be administered.

*Temozolomide*

Temozolomide (Figure 15) is the methyl analogue of mitozolomide, a chloroethylimidazotetrazinone which has been evaluated in phase I and phase II clinical trials. Mitozolomide exhibits some anti-tumour activity in the clinic but trials were terminated because of unpredictable thrombocytopenia. Temozolomide was known to be less potent than mitozolomide in murine anti-tumour tests, and was also less soluble in DMSO. Owing to the limited solubility of mitozolomide and temozolomide, a product formulated in DMSO was used in i.v. administration with temozolomide formulated at a concentration of $30\ mg\ ml^{-1}$. It was not possible to obtain an i.v. $LD_{10}$ in the mouse with the DMSO formulation, however an $LD_{10}$ estimate was obtained by intraperitoneal (i.p.) administration of a suspension. Since it was known that temozolomide is less toxic than mitozolomide and the recommended phase II dose for mitozolomide was $90-100\ mg\ m^{-2}$, it was decided that $50\ mg\ m^{-2}$ would be a safe starting dose for the temozolomide phase I trial. DMSO proved to be an unpopular choice of co-solvent with the nursing staff as the patients exhaled the metabolite dimethylsulphide, which had a characteristic unpleasant odour!! From previous experience, this became a problem when the total volume administered reached 20 ml of DMSO. It was decided that a maximum 15 ml dose of DMSO would be administered i.v., limiting temozolomide dose escalation to $200\ mg\ m^{-2}$. At this dose patients were also given an oral formulation and the measured bioavailability was virtually 100% with a rapid adsorption. Further dose escalations were conducted with an oral formulation and there was no evidence of dose dependency in the pharmacokinetics. Following the determination of the MTD the administration schedule was switched to daily for five consecutive days, and it was on this schedule that activity against gliomas was evident (Stevens and Newlands, 1993). The results from phase II trials against glioma and melanoma are currently under review, and it is hoped to commence further trials with a view to marketing in the near future.

**Figure 14** Chemical structure of V489.

*4-Hydroxyanisole*

4-Hydroxyanisole (Figure 16) had previously undergone phase I clinical trial (Rustin *et al.*, 1992), but it was decided to attempt to deliver total intravenous doses of up to 35 g. The drug's solubility in ethanol enabled this to be used as the vehicle with a final concentration of $600\ mg\ ml^{-1}$, which could be diluted before administration in 0.9% sodium chloride injection. The restraining factor with this formulation was the absolute volume of ethanol that could be infused per hour, which was limited by the rate of ethanol metabolism. If the infusion rate exceeded the metabolic rate it would result in the patients becoming intoxicated, which only happened on a few occasions. Hepatotoxicity was observed during the phase I trial but there was no evidence that this was directly related to the formulation's ethanol content.

### Discussion

The examples above graphically illustrate the many and varied problems associated with the pharmaceutical development of new anti-cancer agents. In the majority of cases it was possible to develop adequate formulations that permitted trials to determine the agent's possible clinical utility. How-

**Figure 15** Chemical structure of temozolomide.

**Figure 16** Chemical structure of 4-hydroxyanisole.

ever, in several cases, despite intense research, development of a suitable formulation proved impossible and some very active compounds were excluded from clinical trials. Poor water solubility and inadequate chemical stability were the main reasons hindering the design of appropriate formulations. New formulation approaches to solubilise and stabilise drugs are therefore urgently needed, and this requires increased basic pharmaceutical research and an infusion of new ideas and approaches. In addition, medicinal chemists must realise the possible pharmaceutical limitations of putative agents and attempt to design appropriate *in vivo* friendly analogues. Many recent compounds with interesting antitumour activity are also very potent (e.g. bryostatin and carzelesin), and as such require special consideration. Not only will suitable formulations need to be developed, but also much thought and attention must be given to handling procedures, clean-up procedures and analytical methods. These must be sufficiently sensitive to monitor effectiveness of clean-up and also the potential of worker exposure when the formulation is produced on a large scale. Special techniques will also be required to minimise loss of these agents by adsorption during administration.

During the past several years a large number of potential new compounds have been developed under the auspices of the JFWP. The JFWP has been the medium for an extensive exchange of pharmaceutical knowledge between CRC, EORTC and NCI and has facilitated the resolution of several formulation problems. It is essential that high quality standards are maintained and further sharpened with sufficient data acquired during development to permit acceptance by the regulatory authorities. Such a move would be another step in speeding the development process from putative agent to useful product. Much has been achieved; however much also remains to be done. Hopefully the JFWP will play an important role in this effort.

**Acknowledgements**
We thank Drs S Burtles, JP Davignon, BW Fox, WR Vezin and OC Yoder for their valuable support to the work of the JFWP since its establishment in 1987.

**References**

ANDERSON WK AND COREY PF. (1977). Synthesis and antileukemic activity of 5-substituted 2,3-dihydro-6,7-bis(hydroxymethyl)-1H-pyrrolizine diesters. *J. Med. Chem.*, **20**, 812–818.

BUCKNALL RA, MOORES H, SIMMS R AND HESP B. (1973). Antiviral effects of aphidicolin, a new antibiotic produced by *Cephalosporium aphidicola*. *Antimicrob. Agents Chemother.*, **4**, 294–298.

BRUNDRET KM, DALZIEL W AND HESP B. (1972). X-ray crystallographic determination of the structure of the antibiotic aphidicolin: a tetracyclic diterpenoid containing a new ring system. *J. Chem. Soc.*, **18**, 1027–1028.

DAVIGNON JP, SLACK JA, BEIJNEN JH, VEZIN WR AND SCHOEMAKER TJ. (1988). EORTC/CRC/NCI Guidelines for the formulation of investigational cytotoxic drugs. *Eur. J. Cancer Clin. Oncol.*, **24**, 1535–1538.

DE VRIES JD, WINKELHORST J, UNDERBERG WJM, HENRAR REC AND BEIJNEN JH. (1993). A systematic study on the chemical stability of the novel indoloquinone antitumour agent E09. *Int. J. Pharm.*, **100**, 181–188.

EL-SAYED, AA AND REPTA AJ. (1983). Solubilization and stabilization of an investigational antineoplastic drug (NSC-278214) in an intravenous formulation using an emulsion vehicle. *Int. J. Pharm.*, **13**, 303–312.

FLORA KP, STELLA VJ, WAUGH WA, FRISZMAN C, WILSON J, VISHNUVAJJALA B AND SMITH T. (1993). Bryostatin 1 (NSC-339555). Development of a dosage form for clinical trials. *Proc. Am. Assoc. Cancer Res.*, **34**, 365.

GABRIELSEN B, MONATH TP, HUGGINS JW, KEFAUVER DF, PETTIT GR, GROSZEK G, HOLLINGSHEAD M, KIRSI JJ, SHANNON WM, SCHUBERT EM, DARE J, UGARKAR B, USSERY MA AND PHELAN MJ. (1992). Antiviral (RNA) activity of selected amaryllidaceae isoquinoline constituents and synthesis of related substances. *J. Natl Prod.* **55**, 1569–1581.

GIBSON M, DENHAM AJ, TAYLOR PM AND PAYNE NI. (1990). Development of a parenteral formulation of trimelamol, a synthetic s-triazine carbinol containing cytotoxic agent. *J. Parenter. Sci. Technol.*, **44**, 306–313.

IWASAKI S, KOBAYASHI H, FURUKAWA J, NAMIKOSHI M, OKUDA S, SATO Z, MATSUDA I AND NODA T. (1984). Studies on macrocyclic lactone antibiotics. VII. Structure of a phytotoxin Rhizoxin produced by *Rhizopus chinensis*. *J. Antibiot.*, **37**, 354–362.

JACKSON C, CRABB TA, GIBSON M, GODFREY R, SAUNDERS R AND THURSTEN DE. (1991). Studies on the stability of trimelamol, a carbinolamine-containing antitumour drug. *J. Pharm. Sci.*, **80**, 245–251.

JUDSON IR, CALVERT AH, RUTTY CJ, ABEL G, GUMBRELL LA, GRAHAM MA, EVANS BD, WILMAN DE, ASHLEY SE AND CAIRNDUFF F. (1989). Phase I trial and pharmacokinetics of trimelamol (N2,N4,N6-trihydroxymethyl-N2,N4,N6-trimethylmelamine). *Cancer Res.*, **49**, 5475–5479.

JUDSON IR, CALVERT AH, GORE ME, BALMANNO K, GUMBRELL LA, PERREN T AND WILTSHAW E. (1991). Phase II trial of trimelamol in refractory ovarian cancer. *Br. J. Cancer.*, **63**, 311–313.

KELLY RC, GEBHARD I, WICNIENSKI N, ARISTOFF PA, JOHNSON PD AND MARTIN DG. (1987). Coupling of cyclopropapyrroloindole (CPI) derivatives. The preparation of CC-1065, ent-CC-1065 and analogues. *J. Am. Chem. Soc.*, **109**, 6837–6838.

LI LH, KELLY RC, WARPEHOSKI MA, McGOVERN JP, GEGHARD I AND DEKONING TF. (1991). Adozelesin, a selected lead among cyclopropylpyrroloindole analogs of the DNA-binding antibiotic, CC-1065. *Invest. New Drugs*, **9**, 137–148.

OOSTVEEN EA AND SPECKAMP WN. (1987). Mitomycin analogs. I. Indoloquinones as (potential) bisalkylating agents. *Tetrahedron*, **43**, 255–262.

PETTIT GR, HERALD CL, DOUBEK DL AND HERALD DL. (1982). Isolation of bryostatin 1. *J. Am. Chem. Soc.*, **104**, 6846–6848.

PETTIT GR, SINGH SB, HAMEL E, LIN CM, ALBERTS DS AND GARCIA-KENDALL D. (1989). Isolation and structure of the strong cell growth and tubulin inhibitor combretastatin A-4. *Experientia*, **45**, 209–211.

RUSTIN GJ, STRATFORD MR, LAMONT A, BLEEHEN N, PHILIP PA, HOWELLS N, WATFA RR AND SLACK J. (1992). Phase I study of intravenous 4-hydroxyanisole. *Eur. J. Cancer*, **28A**, 1362–1364.

RUTTY CJ, JUDSON IR, ABEL G, GODDARD PM, NEWELL DR AND HARRAP KR. (1986). Preclinical toxicology, pharmacokinetics and formulation of N2,N4,N6-trihydroxymethyl-N2,N4,N6-trimethylmelamine (trimelamol), a water-soluble cytotoxic s-triazine which does not require metabolic activation. *Cancer Chemother. Pharmacol.*, **17**, 251–258.

SANDS MS, VIOLANTE MR AND GADEHOLT G. (1987). Computed tomographic enhancement of liver and spleen in the dog with iodipamide ethyl ester particulate suspensions. *Invest. Radiol.*, **22**, 408–416.

SESSA C, ZUCHETTI M, DAVOLI E, CALIFANO R, CAVALLI F, FRUSCATI S, GRUMBRELL L, SULKES A, WINOGRAD B AND D'INCALCI M. (1991). Phase I and clinical evaluation of aphidicolin glycinate. *J. Natl. Cancer Inst.*, **16**, 1160–1164.

STELLA VJ, UMPRAYN K AND WAUGH WN. (1988). Development of parenteral formulations of experimental cytotoxic agents. I. Rhizoxin (NSC-332598). *Int. J. Pharm.*, **43**, 191–199.

STELLA VJ, ANDERSON WK, BENEDETTI A, WAUGH WA AND KILLION RB. (1991). Stability of carmethizole hydrochloride (NSC-602668), an experimental cytotoxic agent *Int. J. Pharm.*, **71**, 157–165.

STEVENS MF AND NEWLANDS ES. (1993). From trazines and triazenes to temozolomide. *Eur. J. Cancer*, **29A**, 1045–1047.

TAGLIABUE G, FILIPPESCHI S, HENDRICKS H AND D'INCALCI M. (1992). Antitumour activity of 1,4-bis(2'-chloroethyl)-1,4-diazabicyclo-[2.2.1]heptane dimaleate (Dabis maleate) in M5076 and its subline resistant to cyclophosphamide M5/CTX. *Ann. Oncol.*, **3**, 233–236.

TAGUCHI T AND OHASHI M. (1978). Aphidicolin prevents mitotic cell division by interfering with the activity of DNA polymerase-α. *Nature*, **275**, 458–460.

TORRES-LABANDEIRA JJ, DAVIGNON JP AND PITHA J. (1991). Oversaturated solutions of drug in hydroxypropylcyclodextrins: parenteral preparation of pancratistatin. *J. Pharm. Sci.*, **80**, 384–386.

VAN DER BURG MEL, PLANTING ASTh, STOTER G, McDANIEL C, VECHT ChJ AND VERWEIJ J. (1991). Phase I study of Dabis Maleate given once every three weeks. *Eur. J. Cancer*, **27**, 1635–1637.

VERWEIJ J, PLANTING ASTh, DE BOER M, VAN DER BURG MEL AND STOTER G. (1992). Frequent administration of Dabis Maleate a Phase I study. *Ann. Oncol.*, **3**, 241.

VEZIN WR AND SALOLE EG. (1993). Development and production of cytotoxic drug formulations for Phase I Trials. In *Pharmaceutical Aspects of Cancer Chemotherapy, Topics in Pharmacy*, Vol. 3, Florence AT and Salole EG. (eds) pp. 104–132. Butterworth-Heineman: London.

WALTON MI, SUGGET N AND WORKMAN P. (1992). The role of human and rodent DT-diaphorase in the reductive metabolism of hypoxic cell cytotoxins. *Int. J. Radiat. Oncol. Biol. Phys.*, **22**, 643–647.

WAUD WR, PLOWMAN J, HARRISON Jr SD, DYKES DJ, ANDERSON WK AND GRISWOLD DP. (1992). Antitumour activity and cross-resistance of carmethizole hydrochloride in preclinical models in mice. *Cancer Chemother. Pharmacol.*, **30**, 261–266.